# Preclinical testing of small diameter Descemet membrane endothelial keratoplasty grafts to increase tissue availability

**DOI:** 10.1371/journal.pone.0246516

**Published:** 2021-02-04

**Authors:** Sorcha Ní Dhubhghaill, Alina Miron, Jessica T. Lie, Isabel Dapena, Silke Oellerich, Gerrit R. J. Melles

**Affiliations:** 1 Netherlands Institute for Innovative Ocular Surgery, Rotterdam, The Netherlands; 2 Antwerp University Hospital, Edegem, Belgium; 3 Melles Cornea Clinic Rotterdam, Rotterdam, The Netherlands; 4 Amnitrans EyeBank Rotterdam, Rotterdam, The Netherlands; Singapore Eye Research Institute, SINGAPORE

## Abstract

In this study, we describe a process of preparing, surgically manipulating, and validating a novel “small diameter” 4mm circular Descemet membrane endothelial keratoplasty (DMEK) graft *in vitro*. Three small diameter DMEK grafts can be prepared from a single donor endothelium and could, therefore, potentially expand the donor pool. Prior to clinical use, however, we aimed to examine each step of the process to determine the effect on the endothelial cell loss and whether or not cells retained their capacity to migrate uniformly. For this study, circular small diameter grafts, obtained from twelve corneas of ten donors deemed ineligible for transplantation, were included. Small diameter DMEK graft preparation was successful in all cases (n = 36). Endothelial cell density (ECD), determined in the eye bank on seventeen grafts, showed an average decrease from 2413 (±189) cells/mm^2^ before to 2240 (±413) cells/mm^2^ after preparation. Twenty-four grafts were used to simulate DMEK-surgery *in vitro* and were successfully stained with 0.06% trypan blue, loaded into a straight DMEK-injector, unfolded, positioned, and centered within the circular ~ 4mm descemetorhexis. The estimated % area populated by viable cells on the grafts decreased from on average 92 (±3) % before to 78 (±10) % (n = 4) after *in vitro* surgery. Cells displayed a capacity for uniform cell migration from all edges of the graft (n = 4) when embedded in the 3D hydrogel system. Our data show, that by using an *in vitro* model of DMEK-surgery it was possible to test the 4mm circular DMEK grafts from eye bank preparation to surgical implantation. The cell loss after *in vitro* surgery was comparable with the *in vivo* ECD decline early after DMEK and the capacity of the cells to migrate to potentially cover bare stroma indicates that these small diameter grafts may be a viable clinical option to treat central endothelial disease.

## Introduction

Descemet membrane endothelial keratoplasty (DMEK) is the current standard of care for patients with symptomatic corneal endothelial dysfunction, with some of the best outcomes being seen in cases of Fuchs endothelial corneal dystrophy (FECD) [1]. While this technique represents an improvement over the classical penetrating keratoplasty (PK) [2] and Descemet stripping (automated) endothelial keratoplasty (DSAEK) [3], it is limited by the 1:1 endothelial donor to recipient ratio, though the stroma may be repurposed for anterior corneal use [4, 5]. In an effort to address this and at the same time trying to possibly reduce the antigen load of the transplanted tissue, Quarter-DMEK was developed, where four patients with central FECD could be treated using a single donor endothelium [6–10]. Clinically, Quarter-DMEK grafts perform well, with similar best corrected visual acuity (BCVA) to conventional DMEK, though the endothelial cell density (ECD) is lower [9], which may be due to the shape mismatch between a round descemetorhexis and a triangular graft. These bare stromal areas require migration of endothelial cells to clear the cornea which could be cause of the reduced ECD [7].

Donor independent strategies for central FECD have also been explored. In these approaches, known as ‘Descemet stripping only’ (DSO) or ‘Descemetorhexis without endothelial keratoplasty’ (DWEK), a smaller descemetorhexis of 4-5mm is performed, removing both the endothelium and associated guttae [10–15]. In successful cases, the residual peripheral endothelial cells spread and migrate to close the defect and clear the cornea. While this does avoid the need for donor tissue, the postoperative healing time is longer and the success rate is less than that of gold standard DMEK techniques [16].

A smaller graft that matches a smaller descemetorhexis could, in theory, be combined to marry the best aspects of both techniques. Miniature DMEK grafts or bare Descemet membrane transplants have been used previously to treat stromal hydrops [17, 18] or to promote endothelial cell migration after manual removal of diseased central corneal endothelial cells from the recipient cornea [19, 20]. While it was technically challenging, it was still feasible to place the patch in the correct place and resulted in clinical improvement. The technique, however, has not been applied to FECD and the effect of preparing and manipulating such grafts on the endothelium is not known.

In this study, we describe a process of preparing and surgically testing small diameter DMEK grafts *in vitro*. The aim was to evaluate not only the feasibility of the surgery but also the effect on endothelial cell density, viability, and migration capacity.

## Materials and methods

### Corneas

Human postmortem corneas, that were deemed ineligible for transplantation, but which had an intact and viable endothelial cell layer, were obtained from Amnitrans EyeBank Rotterdam (www.amnitrans.nl, contact: info@niios.com). Small diameter DMEK grafts were prepared from twelve corneas of ten donors (mean age 69 (±9) years; range 57–85 years) for a total of 36 grafts. The average storage time prior to graft preparation was 13 (±6) days (range 4–21 days) and average ECD 2500 (±230) cells/mm^2^ (range 2100–3000 cells/mm^2^) (Table 1).

**Table 1:** Basic donor demographics of corneas used for small diameter DMEK graft preparation.

Donor Information	
Number of corneas (donors)	12 (10)
Gender	
Female	5
Male	5
Mean age (±SD), yrs (range)	69 (±9), (57–85)
Mean storage time (±SD), days (range)	13 (±6), (4–21)
Mean ECD (±SD), cell/mm^2^ (range)	2500 (±230), (2100–3000)
Rejection reason for corneas (donors)	
Virology	6 (5)
Guttae	1 (1)
Poor endothelium quality (e.g. IOL scars, low ECD, loose roll)	3 (2)
Outdated tissue	2 (2)
Cause of death for corneas (donors)	
Respiratory	4 (3)
Circulatory	2 (2)
Unknown/Others	4 (4)
Cardiovascular	2 (1)

*Mean storage time = time between death and evaluation of the Micro-DMEK at day 0; SD = standard deviation; yrs = years; ECD = endothelial cell density.

All donors had stated to have no objection to transplant-related research and the study adhered to the tenets of the Declaration of Helsinki. No institutional review board approval was obtained as under national regulation no approval is required for this research if no extra procedure was performed to obtain the samples and donors had consented to having the samples used for research purposes (https://www.ccmo.nl/onderzoekers/soorten-onderzoek/niet-wmo-onderzoek/onderzoek-met-lichaamsmateriaal).

### Small diameter Descemet membrane endothelial keratoplasty graft preparation

**Fig 1:**
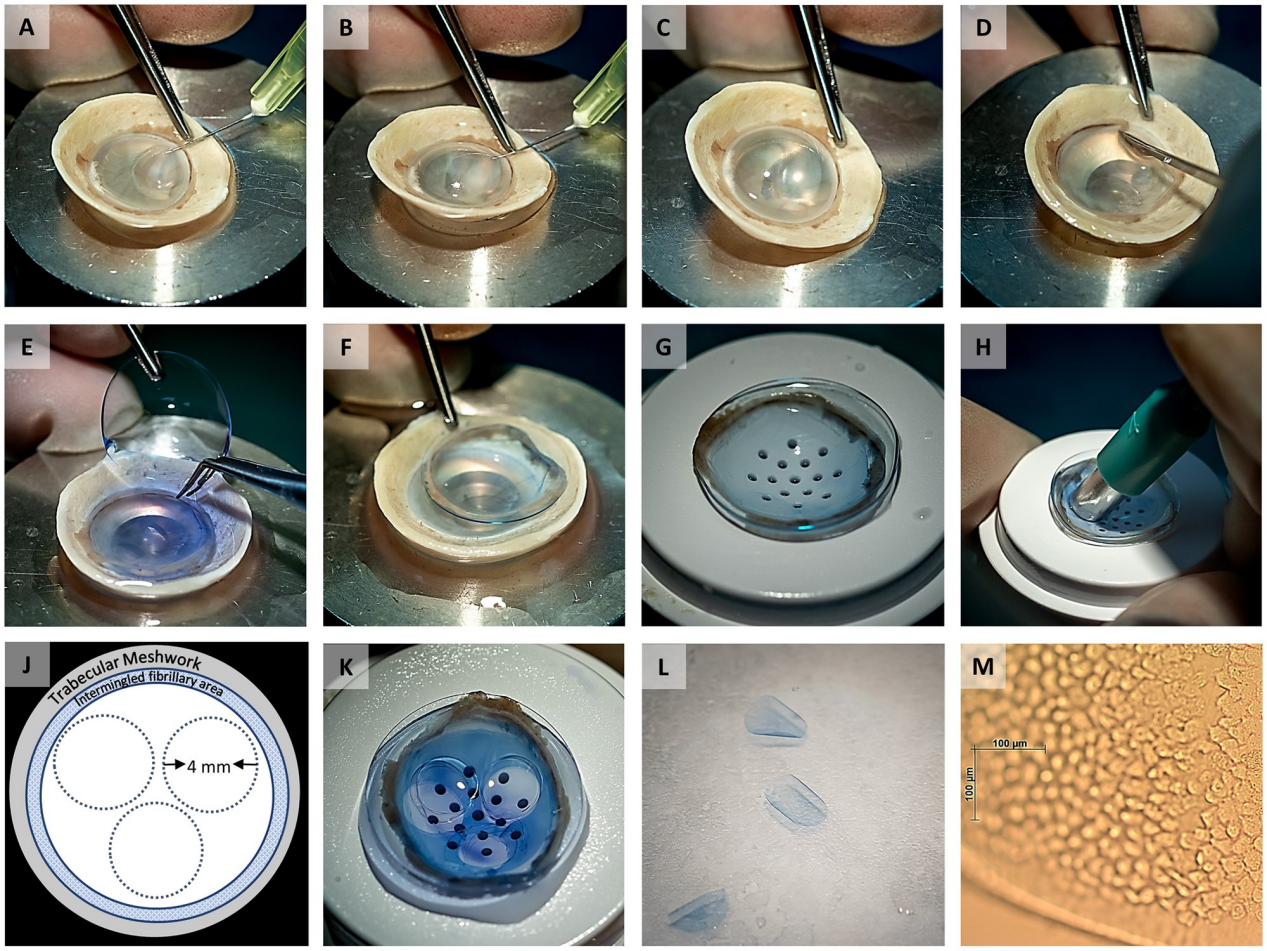
Image collage showing the different steps of preparing three small diameter grafts from one donor cornea. (A) Local DM separation from the stoma by hydro-dissection using a bent 30G needle. (B, C) Separation of the DM along with the corneal endothelium by a bubble spanning the full diameter of the cornea. (D) Complete detachment of the peripheral DM with its adjacent TM using a hockey stick blade. (E) The anterior remnant was replaced on a soft contact lens. (F, G) Transfer of the soft contact lens holding the DM with its TM to a punch block. (H) Preparation of three small diameter grafts by carefully punching out the grafts using a 4 mm diameter biopsy punch. (J) Schematic representation of the trephination pattern for the three grafts. (K, L) Remaining part of the DM sheet after punching out three 4mm grafts and the three resulting grafts. (M) Light microscopy image of a graft showing the endothelial cells on the graft and a very thin denuded band along the graft edge caused by trephination.

Small diameter DMEK donor tissue preparation was performed by a single experienced eye bank technician (JL). Corneoscleral buttons were excised and stored in organ culture medium at 31°C (CorneaMax, Eurobio, Courtaboeuf, France) until graft preparation from whole donor globes obtained less than 24 hours postmortem. The corneoscleral buttons were mounted endothelial side up on a custom-made holder with a suction cup (DORC International, Zuidland, The Netherlands). The Descemet membrane (DM) was separated from the stroma by using a hydro-separation technique using a bent 30G needle (BD Microlance, Drogheda, Ireland) inserted just underneath the DM layer bevel up until the bevel was completely inserted. A small amount of 0.9% physiological salt solution (BSS, B. Braun, Melsungen, Germany) was injected in order to separate DM from the stroma (Fig 1A). Additional physiological salt solution was injected with increased pressure aiming to establish a bubble spanning the full diameter of the cornea (Fig 1B and 1C). Throughout the hydro-separation of the DM from its underlying stroma, the endothelium surface was kept moist by regularly applying BSS solution. After the hydro-dissection, the peripheral DM with its adjacent trabecular meshwork (TM) was loosened over 360 degrees by using a hockey stick blade (Fig 1D) and the anterior remnant was replaced by a soft contact lens (Fig 1E) [4]. The soft contact lens supporting the DM still attached to the TM was placed on a punch block (Network medical products, Ripon North Yorkshire, UK) (Fig 1F and 1G). Attachment of the TM prevented the tissue from scrolling and facilitated further handling. Subsequently, the three grafts were carefully punched out by using a 4mm diameter biopsy punch (Kai Europe GmbH, Solingen, Germany) (Fig 1H, 1J and 1K). Small diameter DMEK grafts were stored in organ-culture medium until the time came for further analysis or *in vitro* surgery (Fig 1L). Endothelial cell density was calculated centrally on the corneas before preparation and on the grafts after preparation using the fixed-frame method by using at least three frames per cornea and graft, respectively (Fig 1M). Post-preparation ECD counts were available for 17 small diameter DMEK grafts with sufficient image quality.

### *In vitro* surgery

**Fig 2:**
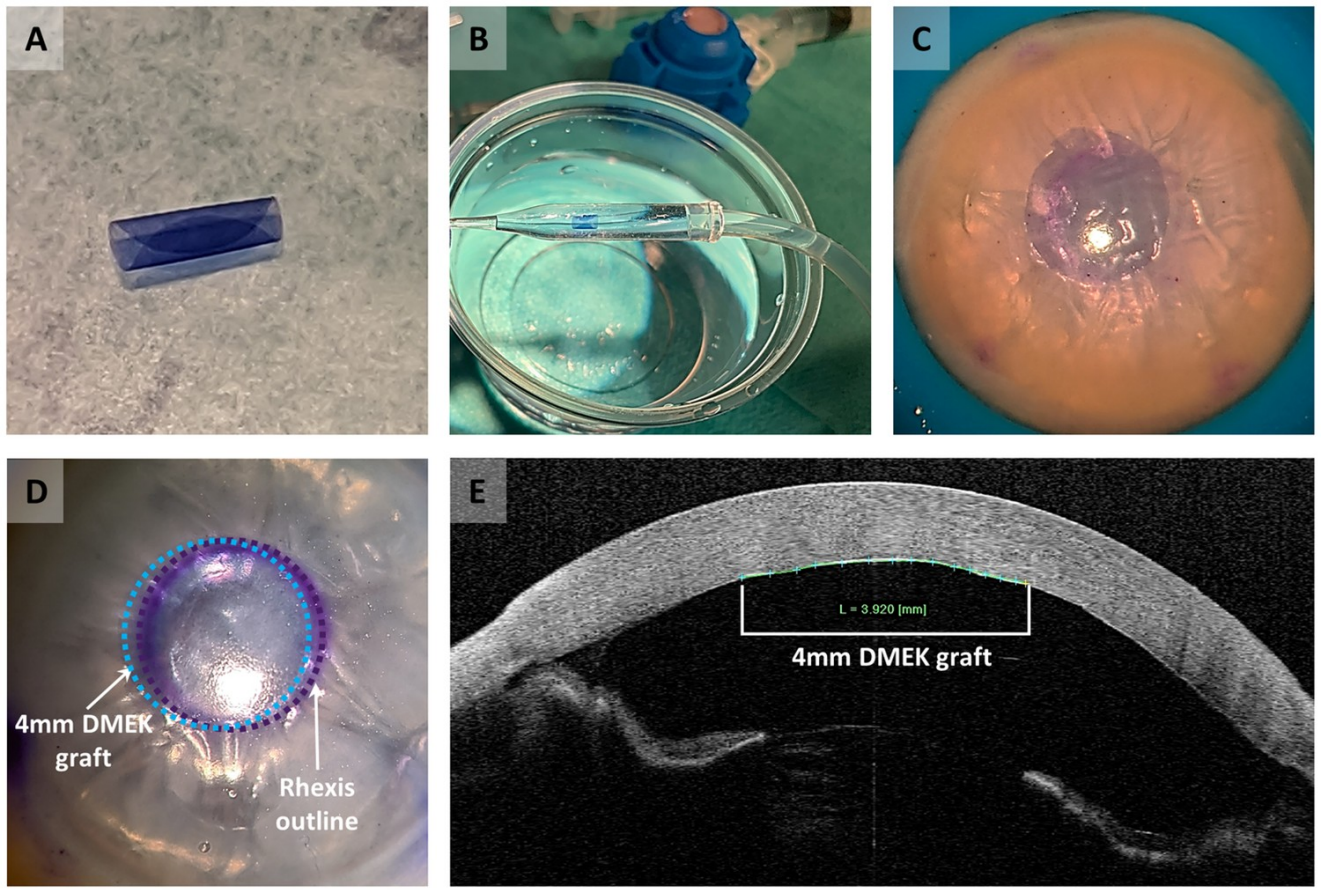
*In vitro* surgery performed with the small diameter DMEK graft. (A) Graft staining with 0.06% trypan blue. (B) Graft loading into a straight DMEK injector. (C) Descemetorhexis performed on a cornea mounted on an artificial anterior chamber. (D) Graft unfolding and positioning in the descemetorhexis area performed in a whole globe. Purple dashed line indicates the outline of the descemetorhexis, and the blue dashed line indicates the position of the 4mm DMEK graft. (E) AS-OCT graft imaging after 30–45 minutes of a fully pressurized anterior chamber.

*In vitro* surgery for 24 of the 4mm DMEK grafts was performed in a manner similar to conventional DMEK [21], with some modifications. All grafts were prepared by staining them twice with 0.06% trypan blue for three minutes (Fig 2A). Grafts were then loaded into a straight DMEK injector (Geuder DMEK injector, Heidelberg, Germany) (Fig 2B). Twenty small diameter DMEK grafts were used during the optimization steps using two types of anterior chamber set ups. The first experiments were performed using a donor corneoscleral button mounted on an artificial anterior chamber. The small graft size and deep chamber, however, made modelling the surgery very difficult. This was made easier by using a flexible thermoplastic material (Parafilm, Bemis Co, USA) to simulate the function of the iris in DMEK surgery. The artificial chamber was primed with balanced salt solution and the film was stretched over it, followed by locking the corneoscleral button in place. Adjusting the pressure in the anterior chamber allowed the surgeon to shallow or deepen the chamber as needed and it was possible to position the graft in the Descemetorhexis (Fig 2C). While this partitioned anterior chamber was very helpful for practicing the maneuvers, the endothelium can adhere to the thermoplastic film and be damaged. To better model the true surgical situation, the final round of experiments was therefore, performed on whole globes (n = 4). The allowed better control of the iris and anterior chamber dept than the model setup.

The globes used were also obtained from Amnitrans EyeBank Rotterdam and had been deemed ineligible for transplantation. Globes were stabilized on a suction support device (DALK/PLK Holder, DORC International). The intraocular pressure could be adjusted by increasing or decreasing the aspiration, fixating the globe. A 4mm circular guiding mark was colored using gentian violet and then stamped on the anterior surface of the cornea. A 3mm self-sealing corneal incision and three 1mm paracentesis incisions were created at the limbus. Air was then injected into the anterior chamber and a Descemetorhexis, slightly larger than the 4mm guiding ring, was created. The graft was then injected into the anterior chamber ensuring that it retained its orientation and that the pressure remained soft to prevent reflux. The anterior chamber was kept relatively shallow during the surgical maneuvers.

Once the graft was centered in the Descemetorhexis, air was slowly injected under the graft until the anterior chamber was completely full (Fig 2D). Full air fill was maintained for 30–45 minutes and imaging was performed using an anterior segment optical coherence tomographer (AS-OCT) (CASIA SS-1000 OCT, Tomey GmbH, Erlangen, Germany) (Fig 2E). At the end of the air-fill period, the corneoscleral rims were carefully excised and transferred endothelial side-up in a glass jar filled with BSS. By gently moving the corneal remnant through the liquid, the graft came loose and, with a glass pipette, could be transferred onto any support and subjected to further biological analysis.

### Graft viability

Calcein-AM was used to examine the cell viability of the grafts both before (i.e. immediately after preparation) and after *in vitro* surgeries. 100 μl of 400 μM Calcein-AM (Sigma-Aldrich Chemistry BV, Zwijndrecht, The Netherlands) in phosphate-buffered saline (PBS) was added directly to grafts that were flattened on silane-coated glass slides. After a 45-minute incubation period at room temperature and one more PBS washing step, fluorescence images were taken (AxioVert.A1 microscope with AxioCam ERc 5s stand-alone functionality camera (Zeiss, Oberkochen, Germany)), and the level of cellular fluorescence was determined with ImageJ using the thresholding method.

### Cell migration study

Four small diameter DMEK rolls were successfully unfolded endothelial-side-up on FNC-coated (fibronectin, collagen, and albumin coating mix, Athena ESTM Baltimore, MD, USA) glass coverslip and evaluated *in vitro* in order to examine the cellular migration behavior. One graft was accidently unfolded endothelium-side-down on the FNC-coated glass and was excluded from the study. The unfolding of all the grafts was performed in a “no-touch” manner by dropping organ-culture medium onto the graft until complete unfolding. Each glass coverslip, which supported one graft, was transferred to a 24-well plate and embedded into the thermoresponsive gel matrix as described previously [22]. Grafts were cultured in a humidified atmosphere at 37°C and 5% CO_2_ for up to 2 weeks. Medium was refreshed every 2–3 days. Grafts were photographed daily with an AxioVert.A1 microscope to examine cell morphology and cell migration. The recovery of the grafts after cultivation was performed by gradually cooling the gel below the sol-gel transition temperature (<20 °C) using low-temperature PBS as the low transfer medium [20].

Cell monolayer integrity was evaluated after gel removal by immunohistochemistry. Two samples were stained for the expression of zonula occludens-1 (ZO-1) according to a protocol that has been described previously [22, 23].

## Results

### Graft preparation

**Fig 3:**
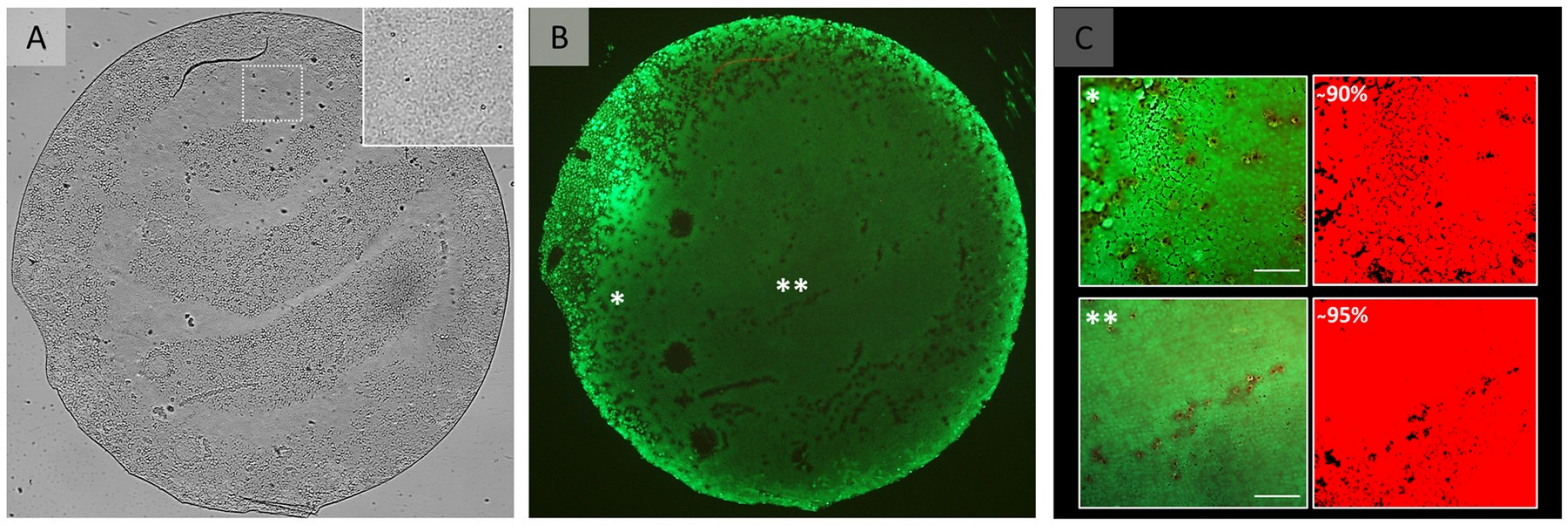
Graft viability after preparation. (A) Light microscopy image of a flattened 4 mm DMEK graft. The insert in the right top corner represents an area on the graft (white square) that may be perceived as a bare area in the overview image but is populated by cells. Please note that areas on the graft that may be perceived as bare areas in are artefacts from the mounting the flat tissue and keeping the graft moist to avoid tissue drying during imaging, which in turn causes some areas to be out of focus. (B) Fluorescence microscopy image of the same graft showing Calcein-AM staining for cell viability. The fluorescence image in (B) shows that all those areas that appeared to be devoid of cells in (A) are indeed covered by viable cells. (C) Higher magnification images from the areas marked by * and ** in overview image (B) to illustrate the viable cells (green) and the corresponding segmentation images (red). Scale bar: 100 μm.

Preparation was successful for all 12 corneas and resulted in 36 grafts of 4mm diameter (Fig 1). All grafts showed endothelial cells up to the graft edge with only a small outer band of cells becoming depleted due to the trephination (Fig 1M). Cells showed the typical endothelial cell morphology and no micro-fibrillar arrangements representative for the far corneal periphery were observed. Post-preparation ECD was 2240 (±413) cells/mm^2^ (n = 17) and it was not statistically significant compared to an ECD of 2413 (±189) cells/mm^2^ (P = .141) calculated centrally before preparation on the same donor corneas used for graft preparation. The cell viability assay performed directly after preparation showed that, on average, 92 (±3) % of the graft surface area was covered by viable cells (Fig 3).

### *In vitro* surgery

*In vitro* surgery was performed using 24 of the 4mm DMEK grafts. In all cases the grafts could be successfully positioned centrally in a 4mm descemetorhexis area. It should be noted that the graft was opened slightly differently than in conventional DMEK. It was noted that intracameral direct fluid injection and air bubble unfolding were not helpful surgical maneuvers, given that the grafts responded by moving too freely around the anterior chamber. Unfolding and centration were instead achieved by soft taps and strokes with a cannula on the outer corneal surface. Post-surgery OCT imaging confirmed complete adherence of the grafts in all cases.

**Fig 4:**
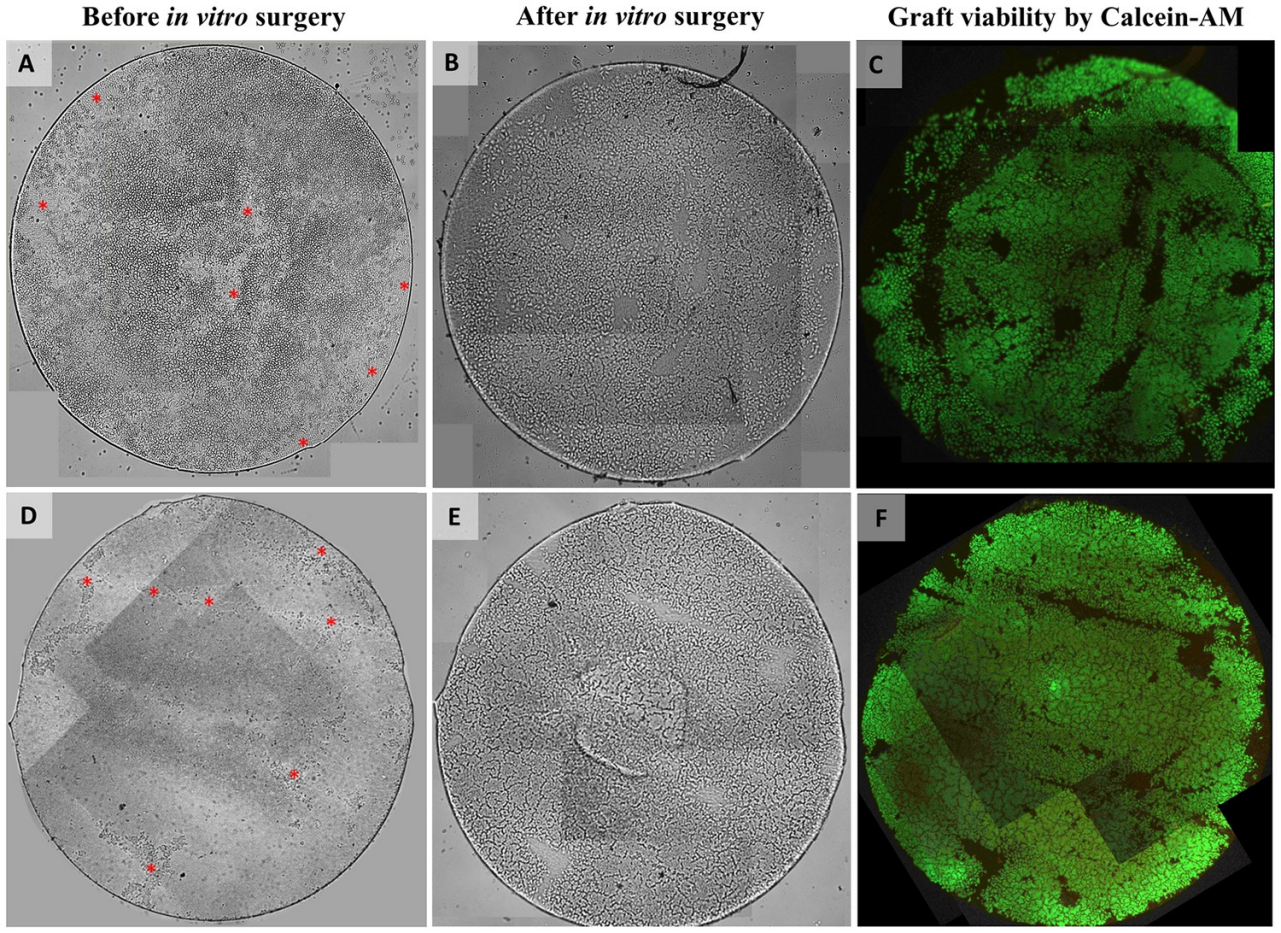
Light and fluorescence microscopy images of two 4mm DMEK grafts before and after *in vitro* surgery. (A, D) Light microscopy images of the grafts before *in vitro* surgery already showing some areas denuded of cells (red asterisk mark) directly after preparation which is probably related to the low-quality of the corneas ineligible for transplantation that were used for preparation. (B, E) Light microscopy images and (C, F) fluorescence microscopy images of the grafts after *in vitro* surgery showing again the same areas devoid of cells in addition to some other small areas on the graft that do not show any Calcein-AM fluorescence signal (indicative for the presence of viable cells). Note that one image tile each if missing in (C, F). The graft surface area within these missing image tiles was calculated (0.7% and 1.9%, respectively) and cell viability percentages were corrected for the missing graft area to avoid a potential overestimation.

For *in vitro* surgeries performed in globes, assessment of cell viability by Calcein-AM assay showed that the estimated average % area populated by viable cells on the grafts was 78 (±10) % (n = 4) after *in vitro* surgery. This would correspond to an average decline of 14% (±5) in viable cell area compared to the 92 (±3) % surface area covered by viable cells determined directly after graft preparation (Fig 4).

### Cell migration study

**Fig 5:**
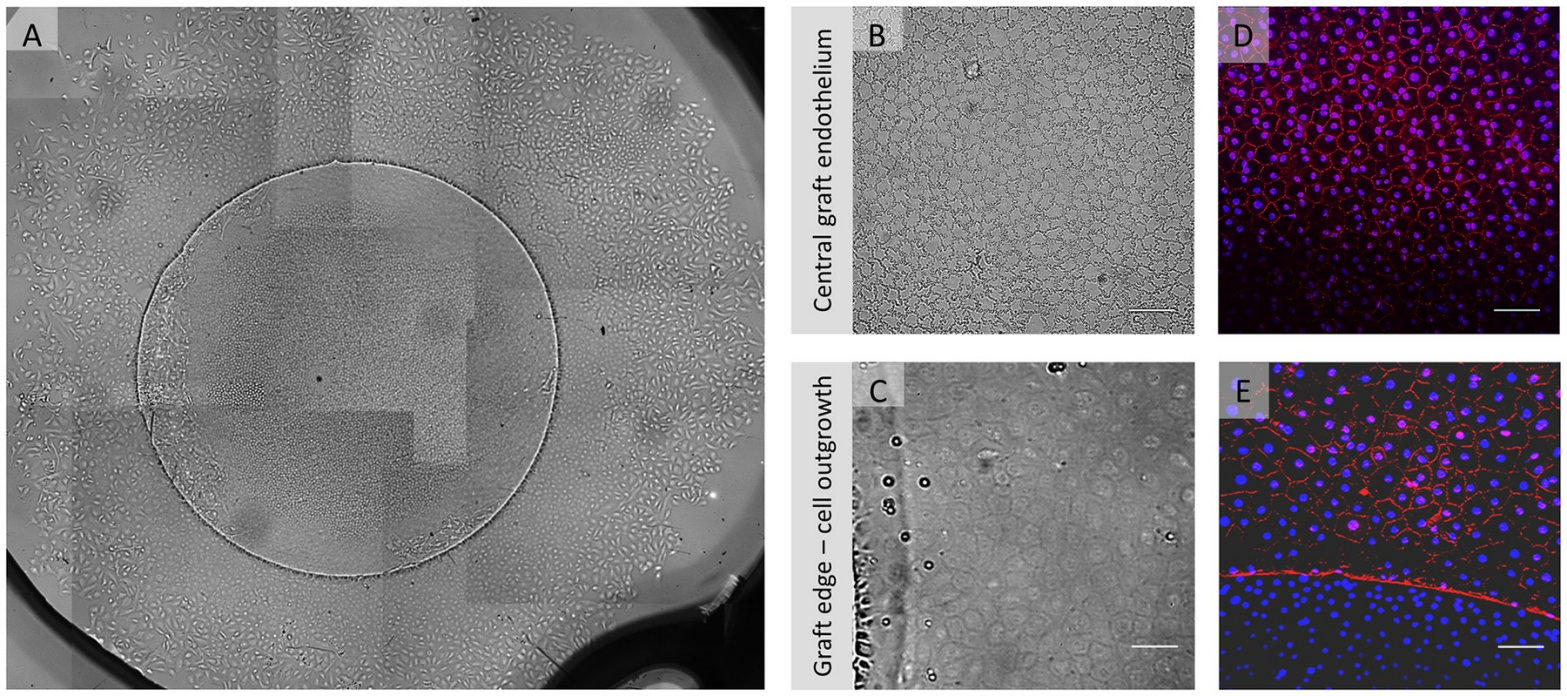
Example of *in vitro* endothelial cell migration from a gel-cultured 4mm graft. (A) Light microscopy overview collage (50x magnification) of a 4 mm DMEK graft after 17 days in gel culture showing uniform cell migration from all around the graft. (B) Light microscopy image showing central graft endothelium and (C) graft edge (bottom of the image) from where migration was directed as a confluent cellular monolayer. (D, E) Fluorescence microscopy images showing expression of ZO-1 (red signal) counterstained with DAPI (blue signal) in the graft center and in the migrated monolayer. The absence of ZO-1 stained cell borders in the lower part of image 5E can be attributed to the fact that cells in this area reside on the graft and are elevated as compared to the new cell monolayer. Therefore, the cells on the graft have an elevation of about 10 μm (on the Descemet membrane) when compared to the migrated cells on the glass cover slide and therefore appear out of focus. Scale bars: 100 μm.

Grafts (n = 4) placed in 3D-gel culture showed uniform cell migration around the entire circular graft edge (Fig 5A and 5C). Endothelial cells appeared densely packed with a homogenous morphology on all grafts (Fig 5B). After gel removal cells showed expression of ZO-1 all across the graft (Fig 5D) and also in the newly formed cell monolayer (Fig 5E).

## Discussion

In this study, we present a new tissue-efficient surgical strategy for the treatment of central FECD by successfully validating the preparation process and *in vitro* surgically protocol for a circular small diameter DMEK graft. With this new application of the small diameter DMEK technique, three circular mini-DMEK grafts with a diameter of 4mm can be obtained from one donor cornea.

The circular shape allows for the graft to be well matched to a 4mm circular descemetorhexis similar to those used in DSO/DWEK procedures [12–21], thereby reducing the bare stroma area that needs to be re-populated postoperatively by endothelial cells. At the same time, harvesting three circular 4mm grafts avoids the far periphery of the corneal endothelium from being included in the graft (Fig 1J). The far peripheral corneal cells of the endothelium are intermingled with collagen fibers which inhibit their capacity to migrate [8, 9]. Thus, by avoiding the periphery in these circular trepanations, it was possible to maintain migration capability from the entire perimeter of the graft.

While surgical handling of such small DMEK grafts could be intuited as prohibitively challenging, the previous work of both Bachmann et al. [17] and Tu [18] indicates that small DMEK grafts can be mobilized effectively in the anterior chamber, even under the visual obstruction of stromal hydrops. Postoperative endothelial cell density was not a main outcome parameter, however, as the primary purpose was to patch ruptured DM so “no-touch” handling was not mandatory. In our surgical testing model, we demonstrated that circular 4mm DMEK grafts can be handled and centered in a circular descemetorhexis in a “no-touch” fashion with minimal loss in cell viability and migration capacity offering the possibility of using this small patch technique for FECD. The model itself also provided an opportunity to practice surgical maneuvers which may help in reducing the learning curve when translating this technique to patients.

One limitation of our analysis is the small number of grafts tested in a globe model while the other grafts were used for technique optimization in a different surgical model. This limits the sample size and thus accuracy of the cell viability analysis. Also, for cell viability analysis grafts could not be evaluated before *and* after surgery, and in addition had to be removed from the globe for imaging which constitutes an additional handling. However, the latter may be expected to have rather decreased than increased the reported cell viability percentage after *in vitro* surgery. With these limitations, the viability estimates are to be considered an extrapolation but they still provide some reassurance prior to applying this technique in a patient’s eye. For future studies, using prolonged Calcein-AM staining to monitor the decrease in cell viability during the entire process from graft preparation to surgery may provide more detailed information on the effect of each handling step [24]. It should also be noted that small-diameter DMEK grafts are not a replacement for full-sized DMEKs, particularly in extensive FECD and bullous keratopathy, where peripheral corneal edema is a prominent feature. They should rather be considered an option for central FECD and a potential alternative, or rescue strategy, for DSO/DWEK. By having a matching shape to the circular descemetorhexis, small diameter DMEK grafts may provide a faster corneal clearance than DSO. After successfully *in vitro* testing of small diameter DMEK grafts from eye bank preparation to surgical implantation, clinical tests will be required to evaluate if small diameter-DMEK can indeed become a viable clinical option to treat central endothelial disease.

## Supporting information

S1 Fig Light microscopy examination of the research-graded human corneas before small dimeter DMEK graft preparation. For cell viability assessment after preparation. For cell viability assessment after preparation in the eye bank, grafts of corneas A, B, C, I and K were used. Grafts used to optimize the learning curve and tested using artificial anterior chamber model were prepared from corneas A-I. The four grafts transferred into globes were prepared from L and K. Migration studies were performed with grafts of corneas B, C and J.
(PDF)
