# Supplementary material for: Preclinical testing of small diameter Descemet membrane endothelial keratoplasty grafts to increase tissue availability
Source: PLoS One. 2021 Feb 4;16(2):e0246516. doi: 10.1371/journal.pone.0246516 (PMC7861447; doi:10.1371/journal.pone.0246516)
Supplement: S1 Fig — For cell viability assessment after preparation. For cell viability assessment after preparation in the eye bank, grafts of corneas A, B, C, I and K were used. Grafts used to optimize the learning curve and tested using artificial anterior chamber model were prepared from corneas A-I. The four grafts transferred into globes were prepared from L and K. Migration studies were performed with grafts of corneas B, C and J. (PDF) [file pone.0246516.s001.pdf]

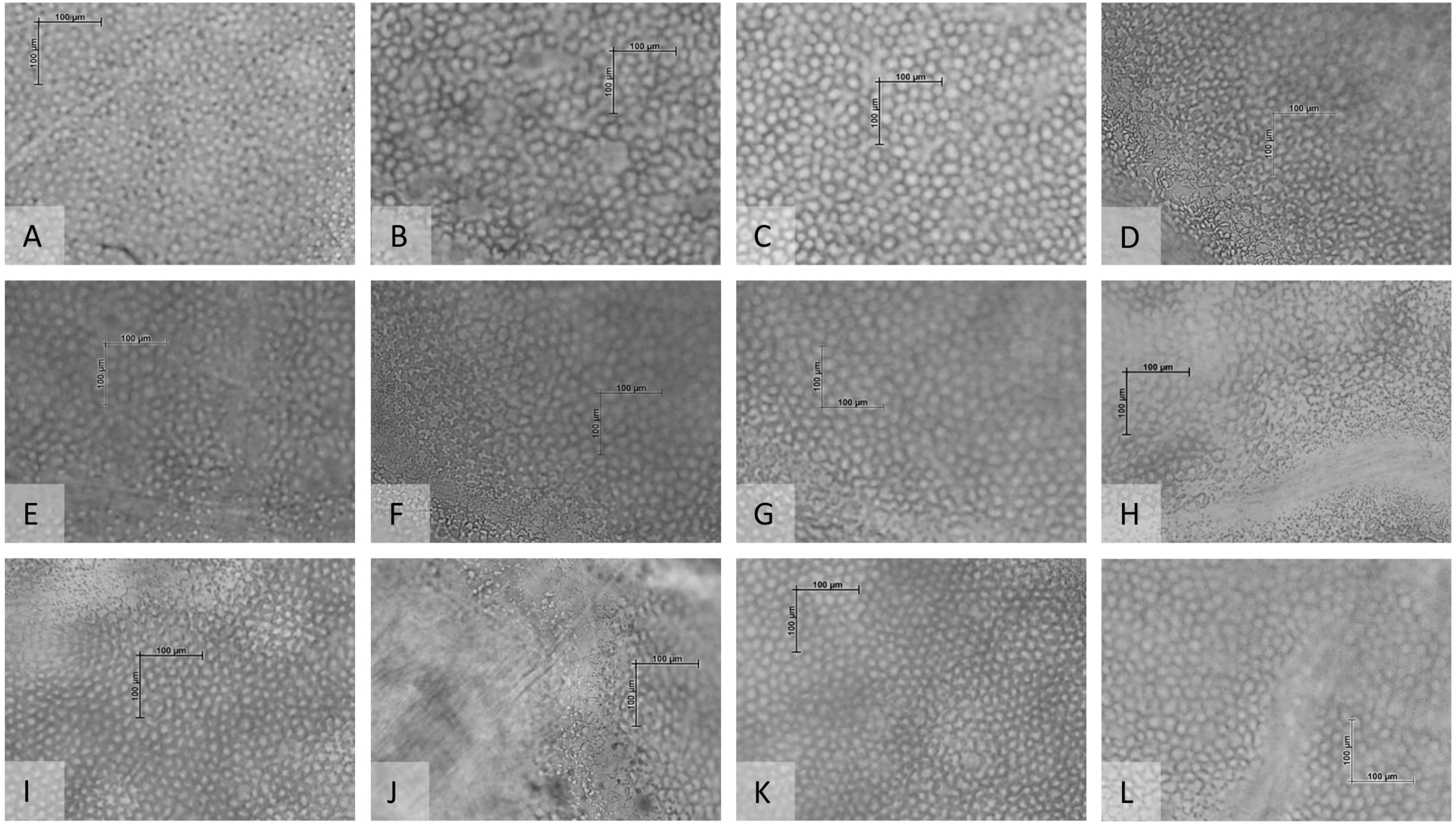

S1 Fig. Light microscopy examination of the research-graded human corneas before small dimeter DMEK graft preparation. For cell viability assessment after preparation in the eye bank, grafts of corneas A, B, C, I, and K were used. Grafts used to optimize the learning curve and tested using the artificial anterior chamber model were prepared from corneas A – I. The four grafts transferred into globes were prepared from corneas L and K. Migration studies were performed with grafts of corneas B, C, and J.
